# Integration analysis identifies MYBL1 as a novel immunotherapy biomarker affecting the immune microenvironment in clear cell renal cell carcinoma: Evidence based on machine learning and experiments

**DOI:** 10.3389/fimmu.2022.1080403

**Published:** 2022-12-14

**Authors:** Tengda Wang, Wengang Jian, Wei Xue, Yuyang Meng, Zhinan Xia, Qinchen Li, Shenhao Xu, Yu Dong, Anli Mao, Cheng Zhang

**Affiliations:** ^1^ Urology Surgery Department, The First Affiliated Hospital of Harbin Medical University, Harbin, China; ^2^ The Fourth Affiliated Hospital, International Institutes of Medicine, Zhejiang University School of Medicine, Yiwu, Zhejiang, China

**Keywords:** MYBL1, immunotherapy, machine learning, prognosis, ccRCC

## Abstract

**Background:**

Previous studies have identified MYBL1 as a cancer-promoting molecule in numerous types of cancer. Nevertheless, the role of MYBL in renal cancer remains unclear.

**Methods:**

Genomic and clinical data of clear cell renal cell carcinoma (ccRCC) was get from the Cancer Genome Atlas (TCGA) database. CCK8, colony formation, and 5-ethynyl-2’-deoxyuridine assay were utilized to evaluate the performance of cell proliferation. Cell apoptosis was detected using the flow cytometric analysis. The protein level of MYBL1 in different tissues was evaluated using immunohistochemistry. A machine learning algorithm was utilized to identify the prognosis signature based on MYBL1-derived molecules.

**Results:**

Here, we comprehensively investigated the role of MYBL1 in ccRCC. Here, we noticed a higher level of MYBL1 in ccRCC patients in both RNA and protein levels. Further analysis showed that MYBL1 was correlated with progressive clinical characteristics and worse prognosis performance. Biological enrichment analysis showed that MYBL1 can activate multiple oncogenic pathways in ccRCC. Moreover, we found that MYBL1 can remodel the immune microenvironment of ccRCC and affect the immunotherapy response. *In vitro* and *in vivo* assays indicated that MYBL1 was upregulated in ccRCC cells and can promote cellular malignant behaviors of ccRCC. Ultimately, an machine learning algorithm – LASSO logistics regression was utilized to identify a prognosis signature based on the MYBL1-derived molecules, which showed satisfactory prediction ability on patient prognosis in both training and validation cohorts.

**Conclusions:**

Our result indicated that MYBL1 is a novel biomarker of ccRCC, which can remodel the tumor microenvironment, affect immunotherapy response and guide precision medicine in ccRCC.

## Introduction

Renal cancer represents a frequent malignancy globally with approximately 4 and 1.9 million new cases and death each year, respectively (1). Among all cases of renal cancer, clear cell renal cell carcinoma (ccRCC) is the leading pathological subtype (2). As a multifactorial multi-factorial disorder, ccRCC is associated with obesity, smoking, dietary habits, environmental exposure, genetic susceptibility, and so on (3). For ccRCC patients at the local stage, surgical resection combined with adjuvant agents is still the mainstay therapy choice (4). Nonetheless, the prognosis is still unsatisfactory for patients in the advanced stage or with distant metastasis (4). Consequently, identifying a novel target for ccRCC diagnosis and treatment is meaningful for clinical application.

The rapid development of bioinformatics analysis has brought researchers great convenience in deeply understanding specific molecules in diseases (5). For instance, Wei et al. revealed that MX2 might be a biomarker indicating sunitinib resistance (6). The MYBL1 has been implicated in multiple diseases. Zhu et al. indicated that MYBL1 was tightly associated with higher endothelial vessel density by inducing the transcriptional activation of ANGPT2, further affecting sorafenib resistance in liver cancer (7). Guo et al. indicated that the O-GlcNAc can regulate MYBL1 expression in an epigenetic modification manner, leading to an aberrant cancer stem cell compartment and cancer progression (8). Brayer et al. demonstrated that the fusion of MYB and MYBL1 contributes to the oncogenic pathway in salivary gland adenoid cystic carcinoma (9). Ramkissoon et al. indicated that MYBL1 also acted as an oncogene factor in glioma (10). Nikolaus et al. revealed that the MYBL1 might be a trigger for autoimmune encephalitis, indicating its role in the disease immune (11). However, there is no previous study focused on MYBL1 in ccRCC.

In this study, we comprehensively the effects MYBL1 produces in ccRCC through bioinformatics analysis and experiments. We noticed a higher level of MYBL1 in ccRCC patients in both RNA and protein levels. Further analysis showed that MYBL1 was correlated with progressive clinical characteristics and worse prognosis performance. Biological enrichment analysis was conducted to identify the biological role of MYBL1 in ccRCC. Moreover, we noticed that MYBL1 can remodel the immune microenvironment of ccRCC and affect the immunotherapy response. *In vitro* and *in vivo* assays indicated that MYBL1 was upregulated in ccRCC cells and can promote cellular malignant behaviors of ccRCC. Meanwhile, the prognosis signature based on the MYBL1-derived molecules showed great prediction ability on patient prognosis.

## Methods

### Public data collection

The open-accessed data on transcriptional profiles and clinical features were all download from The Cancer Genome Atlas (TCGA) database, the KIRC project and the Arrayexpress database, E-MTAB-1980 project. Initial expression profile of ccRCC patients (STAR-Counts form) was collated to a combined matrix (TPM” form) for subsequent analysis. We extracted the survival and clinical information based on the bcr-xml file in TCGA-KIRC. Pan-cancer expression data was obtained from the UCSC Xena website (https://xenabrowser.net/). The limma and affay packages in the R environment were utilized for data preprocessing, consisting of background correction, probe ID annotation, missing value completion and normalization. Extra gene expression information of normal tissue was get from the GETx database. The open-accessed immunohistochemical images of renal cancer were get from The Human Protein Atlas (HPA) database. Baseline features of ccRCC patients enrolled in the analysis were presented in **Tables 1**, **2**. The limma package in the R environment was utilized for DEGs analysis under specific parameters.

**Table 1:** Baseline information of patients in TCGA-KIRC.

Clinical features		Numbers	Percentage (%)
Age	<=65	352	65.5
	>65	185	34.5
Gender	Female	191	35.6
	Male	346	64.4
Grade	G1	14	2.6
	G2	230	42.8
	G3	207	38.5
	G4	78	14.5
	Unknown	8	1.5
Stage	Stage I	269	50.1
	Stage II	57	10.6
	Stage III	125	23.3
	Stage IV	83	15.5
	Unknown	3	0.6
T-stage	T1	275	51.2
	T2	69	12.8
	T3	182	33.9
	T4	11	2.0
M-stage	M0	426	79.3
	M1	79	14.7
	Unknown	32	6.0
N-stage	N0	240	44.7
	N1	17	3.2
	Unknown	280	52.1

**Table 2:** Baseline information of patients in E-MTAB-1980.

Clinical features		Numbers	Percentage (%)
Age	<=65	145	60.4
	>65	95	39.6
Gender	Female	56	23.3
	Male	184	76.7
Grade	G1	42	17.5
	G2	141	58.8
	G3	49	20.4
	G4	6	2.5
	Unknown	2	0.8
T-stage	T1	187	77.9
	T2	18	7.5
	T3	33	13.8
	T4	2	0.8
M-stage	M0	215	89.6
	M1	25	10.4
N-stage	N0	238	99.2
	N1	2	0.8

### Biological enrichment

The clueGO app built in Cytoscape software provides the biological terms for the input molecules identified, as well as an intuitive representation (12). Clusterprofiler in R was used to enrich Gene Ontology (GO) terms (13). Gene Set Enrichment Analysis (GSEA) was utilized to investigate the potential biological differences between two selected groups based on set reference gene sets, including Hallmark gene set (14). The single sample GSEA (ssGSEA) was utilized to quantify the enrichment score based on a specific reference file (15).

### Immune-related analysis

The XCELL, MCPCOUNTER, CIBERSORT, TIMER, EPIC and QUANTISEQ algorithms were utilized for immune microenvironment quantification (16–19). The Immunophenoscore (IPS), an machine learning algorithm from The Cancer Immunome Database (TCIA), was utilized to quantify the IPS score of ccRCC patients based on their transcriptional profile, indicating the response to immunotherapy (20). Meanwhile, the Tumor Immune Dysfunction and Exclusion (TIDE) algorithm was also utilized to assess patients immunotherapy response (21).

### Genomic characterization

Two important immunotherapy markers, tumor mutation burden (TMB) and microsatellite instability (MSI) score were get from the TCGA database. The gene mutation characteristic of MYBL1 in the TCGA database was obtained and visualized based on an online website, https://www.home-for-researchers.com/. The score of the tumor stemness index (mRNAsi) in the TCGA-KIRC project was obtained from the previous study, which was completed using the OCLR machine-learning algorithm (22).

### Nomogram

With the survival and rms packages, a nomogram plot was created based on multiple factors. The calibration and Decision Curve Analysis (DCA) plot were utilized to evaluate nomogram performance.

### Identification of prognosis signature based on machine learning

For TCGA database, all patients were randomly divided into training and internal validation cohorts according to the ratio of 1 to 1. A univariate Cox regression analysis was conducted on the DEGs identified between high and low MYBL1 expression to identify the molecules significantly correlated with patient survival. Then, an machine learning algorithm, LASSO logistic regression, was utilized for variable optimization (23). The optimized variables were then set as the input file for multivariate Cox regression analysis. Ultimately, a prognosis signature was identified with the formula of “Risk score = Expression of A * Coef A + Expression of B * Coef B + … + Expression of X * Coef X”. The E-MTAB-1980 project was used as the external validation cohort.

### Cell culture

Four renal cancer cell lines (A498, ACHN, 786-O and OS-RC-2) and normal cell line HK-2 were laboratory stocks. All cells were routinely cultured using the 10% heat-inactivated fetal bovine serum (37°C with 5% CO_2_) and passaged for three days a time (24).

### RNA isolation and quantitative RT-PCR

Total RNA extraction and steps for qRT-PCR were conducted following our previous study (24). The sequence of primers was as follows: MYBL1, forward primer, 5’-TAGCACTCCACCAGCCATCCTC-3’, reverse primer, 5’-ACCACCATCGTTCAATGAGCCATC-3’.

### Retroviral infection, and transfection

We purchased HBLV-h-MYBL1 shRNA#1-PURO, HBLV-h-MYBL1 shRNA#2-PRUO and HBLV-h-MYBL1-Ctl-PURO from Hanbio. Cell transfection was performed by jetPRIME (Polyplus, NY, USA) referring to the manufacturer’s protocol. We constructed the stably lentivirus-transinfected cells with puromycin (MCE.NJ) to collect the MYBL1 stable-knockdown cells. The sequence used for shRNA were as follow: MYBL1 sh#1, top strand, 5’-GATCCGGACGAGGATGATAAATTACTCGAGTAATTTATCATCCTCGTCCTTTTTTG-3’, bottom strand, 5’-AATTCAAAAAAGGACGAGGATGATAAATTACTCGAGTAATTTATCATCCTCGTCCG-3’; sh#2, top strand, 5’-GATCCGCCATGGAATGCCAATTTACTCGAGTAAATTGGCATTCCATGGCTTTTTTG-3’, bottom strand, 5’-AATTCAAAAAAGCCATGGAATGCCAATTTACTCGAGTAAATTGGCATTCCATGGCG-3’.

### CCK-8 assay

Steps for **CCK-8** were completed following our previous study based on the sh-MYBL1 and control cells (24).

### Colony formation assay

Steps for colony formation assay were completed following our previous study based on the sh-MYBL1 and control cells (24).

### 5-ethynyl-2’-deoxyuridine assay

Steps for EdU assay were completed following our previous study based on the sh-MYBL1 and control cells (24).

### Cell apoptosis assays

Steps for cell apoptosis detection were completed following our previous study based on the sh-MYBL1 and control cells (24). The results were analyzed through FlowJo 6.2 software.

### Xenograft models

Steps for xenograft model assay were completed following our previous study based on the sh-MYBL1 and control cells (Approximately 6 × 10^6^ 786-O cells, MYBL1 sh#Ctrl and sh#1) (24). Animal procedures were performed in line with the Association for Assessment and Accreditation of Laboratory Animal Care and approved by the Animal Care and Use Committee of the First Affiliated Hospital of Harbin Medical University.

### Patient and clinical samples

The study was admitted by the First Affiliated Hospital of Harbin Medical University. ccRCC and adjacent tissue samples were obtained from patients who were aware of the purpose of the study and signed informed consent at the Medical Ethics Committee of First Affiliated Hospital of Harbin Medical University. After the operations of radical nephrectomy, Half of the samples were frozen in liquid nitrogen, and half were embedded in paraffin after being fixed with 4% paraformaldehyde overnight at room temperature until further use.

### Immunohistochemistry

The clinical samples and tumor tissues embedded in paraffin were cut into 5-μm-thick sections. The sections were blocked by 5% goat serum and incubated overnight at 4°C with antibodies against MYBL1 (1:400, Boster, Wuhan, China). We used the DAB (Beyotime, Shanghai, China) system for detection. We chose the three fields in each section and took photos and analyzed the images by ImageJ software.

### Statistical analysis

R and GraphPad Prism 8 software were utilized for all statistical analysis. The statistical threshold of the P value in comparison was 0.05. All the experiments were repeated at least three times.

## Results

### Pan-cancer analysis of MYBL1 and its clinical role in ccRCC

**Figure 1:**
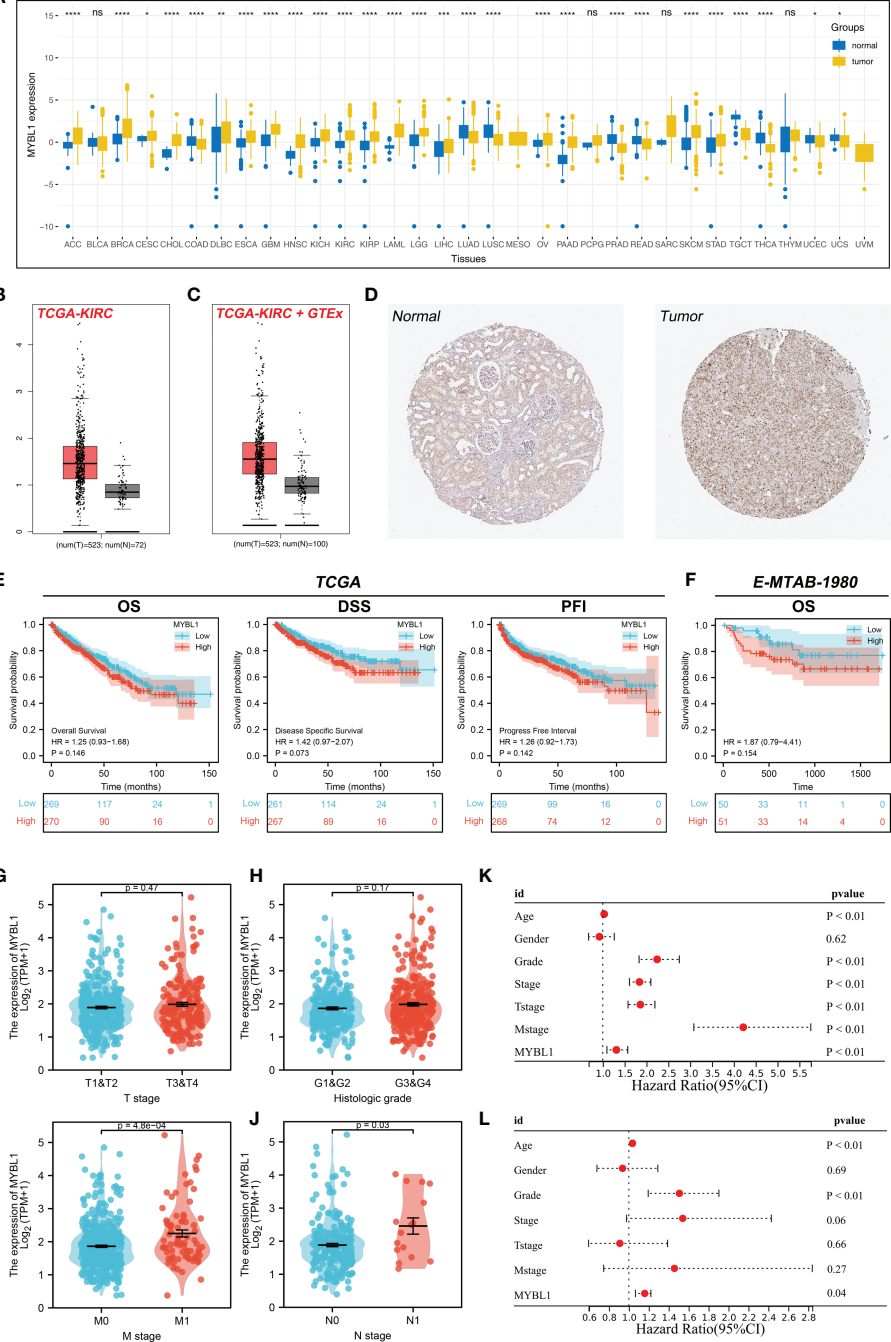
Expression pattern and clinical role of MYBL in ccRCC. **(A)** The landscape of MYBL1 in pan-cancer; **(B)** Expression of MYBL1 in ccRCC tumor and normal tissue based on the TCGA database; **(C)** Expression of MYBL1 in ccRCC tumor and normal tissue based on the TCGA+GTEx database; **(D)** Representative immunohistochemical images of MYBL1 in HPA database; **(E)** Prognosis performance of MYBL1 in TCGA database; **(F)** Prognosis performance of MYBL1 in E-MTAB-1980 database; **(G-J)** Clinical correlation of MYBL1; **(K, L)** Univariate and multivariate analysis of MYBL1. *P < 0.05, **P < 0.01, ***P <0.001, ****P <0.0001, "ns" means P > 0.05.

The flow chart of the whole study was shown in **Figure S1**. The expression landscape of MYBL1 was illustrated in **Figure 1A**, in which MYBL1 showed an abnormal expression pattern in most of the cancers, indicating its important role in cancer development. According to the GTEx and TCGA data, MYBL1 all showed a higher expression level in ccRCC tumor tissue compared with the control normal tissue (**Figures 1B, C**). Moreover, based on the immunohistochemical result from the HPA database, a higher protein level of MYBL1 in renal cancer tissue was observed (**Figure 1D**). Furthermore, we tried to investigate the prognosis role of MYBL1 in ccRCC. Results indicated that MYBL1 might be correlated with worse prognosis performance of ccRCC patients, including overall survival (OS), disease-free survival (DSS) and progression-free survival (PFI) in both TCGA and E-MTAB-1980 cohorts (**Figures 1E, F**). Then, we explored the clinical correlation of MYBL1 in ccRCC patients. No remarkable difference in MYBL1 expression was found in patients with different T-stage and grades (**Figures 1G, H**). However, a significantly higher level of MYBL1 was noticed in patients with worse M- and N-stage, indicating its promoting effect in cancer metastasis (**Figures 1I, J**). Based on univariate and multivariate analyses, MYBL1 was an independent risk factor for ccRCC (**Figures 1K, L**).

### MYBL1 exerts a wide biological regulatory effect in ccRCC

**Figure 2:**
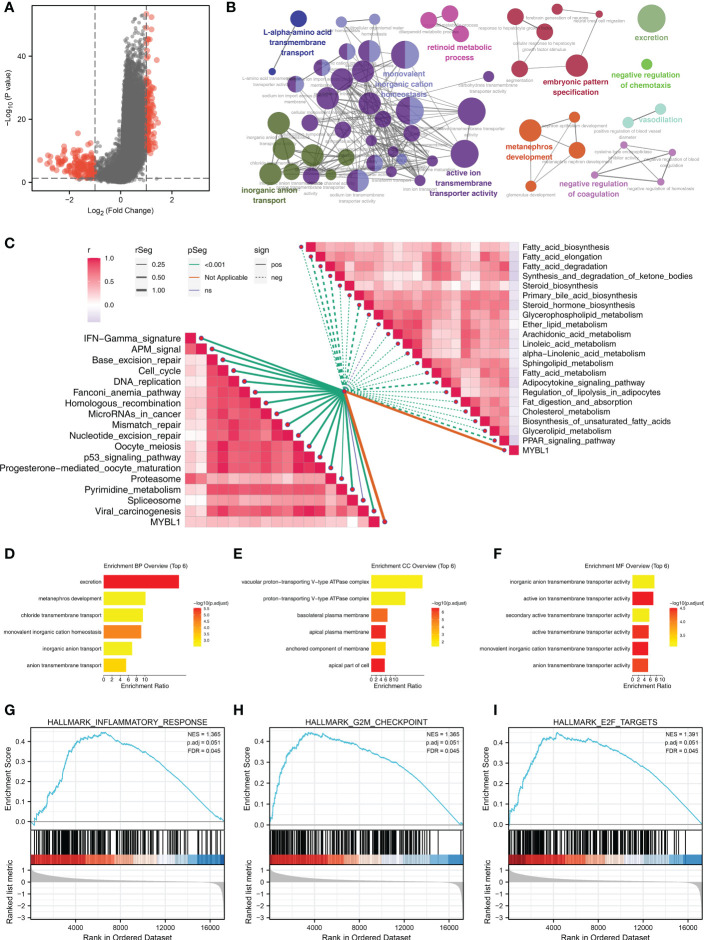
The biological role of MYBL1. **(A)** DEGs between the high and low MYBL1 expression; **(B)** clueGO analysis of DEGs; **(C)** Correlation of the pathways quantified by ssGSEA algorithm; **(D–F)** GO analysis of the DEGs; **(G–I)** GSEA analysis of Hallmark gene set.

A total of 154 downregulated and 136 upregulated DEGs were identified in patients with high and low MYBL1 expression (**Figure 2A**). These DEGs were involved in L-alpha-amino acid transmembrane transport, inorganic anion transport, monovalent inorganic cation homeostasis, retinoid metabolic process, active ion transmembrane transporter activity, metanephros development, embryonic pattern specification, excretion, negative regulation of chemotaxis, vasodilation, negative regulation of coagulation based on clueGO analysis (**Figure 2B**). Moreover, the results of the ssGSEA algorithm indicated that MYBL1 was positively correlated with most immune terms, especially base excision repair, fanconi anemia pathway, and homologous recombination; MYBL1 was negatively correlated with most metabolism terms (**Figure 2C**). GO analysis indicated that for the Biological Process (BP), the top enriched terms were excretion, metanephros development, chloride transmembrane transport (**Figure 2D**). For the Cellular Component (CC) and Molecular Function (MF), the terms were mainly enriched in the transport complex (**Figures 2E, F**). The GSEA analysis of Hallmark gene set was mainly enriched in the terms of the inflammatory response, G2M checkpoint and E2F targets (**Figures 2G–I**).

### MYBL1 can remodel the ccRCC immune microenvironment

**Figure 3:**
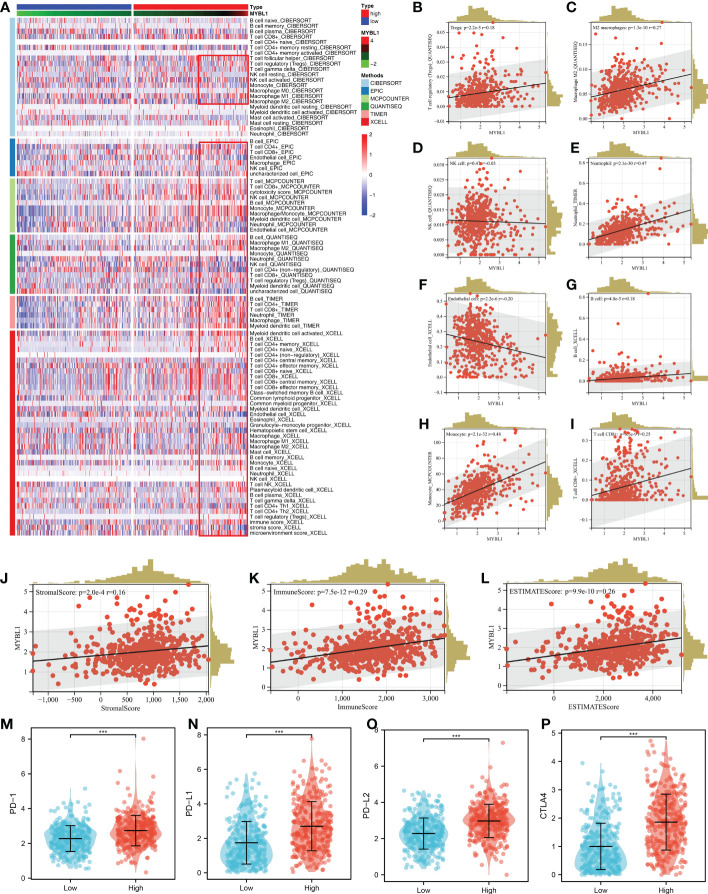
The immune landscape of MYBL1. **(A)** Immune infiltration of MYBL1; **(B–I)** Correlation between MYBL1 and multiple immune cells; **(J–L)** Correlation of MYBL1 and immune score, stromal score and estimate score; **(M–P)** The expression level of key immune checkpoints in patients with high and low MYBL1 expression.

Based on the XCELL, MCPCOUNTER, CIBERSORT, TIMER, EPIC and QUANTISEQ algorithms, we quantified the immune microenvironment of ccRCC samples. A different immune infiltration pattern was observed in patients with high and low MYBL1 expression (**Figure 3A**). Immune correlation indicated that MYBL1 can increase Tregs, M2 macrophages, neutrophils, B cells, monocytes, CD8+ T cells, yet decrease endothelial cells level in the ccRCC microenvironment (**Figures 3B–I**). Moreover, we found that MYBL1 was positively correlated with immune score, stromal score and estimate score (**Figures 3J–L**). Interestingly, we found that the key immune checkpoints PD-1, CTLA4, PD-L1 and PD-L2 all present a high level in patients with higher MYBL1 expression, indicating that MYBL1 might affect the immunotherapy response of ccRCC patients (**Figures 3M–P**).

### Role of MYBL in ccRCC genomic characteristics

**Figure 4:**
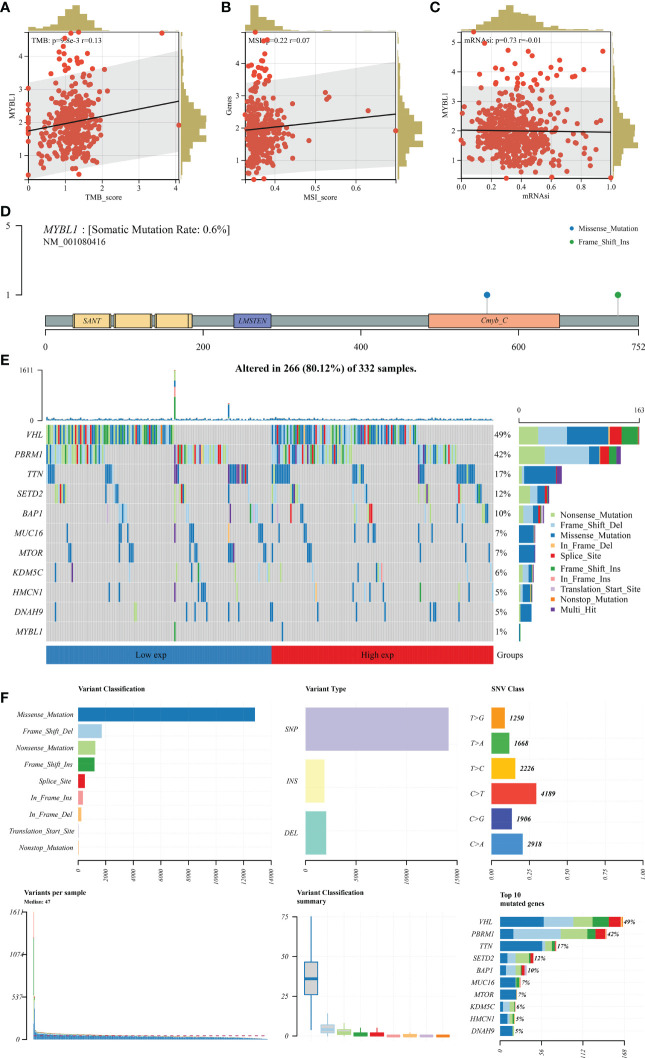
Genomic characteristics of MYBL1. **(A)** Correlation of MYBL1 with TMB; **(B)** Correlation of MYBL1 with MSI; **(C)** Correlation of MYBL1 and mRNAsi; **(D–F)** The mutation characteristics of MYBL1.

TMB and MSI are important markers for cancer immunotherapy and can also indicate genomic instability. A positive correlation was observed in MYBL1 with TMB and MSI (**Figures 4A, B**; TMB: R = 0.13; MSI: R = 0.22). Nevertheless, MYBL1 might has no significant effect on mRNAsi (**Figure 4C**). The genomic mutation characteristics of MYBL1 was shown in **Figure 4D** (0.6% somatic mutation rate). The top five most differentially mutated genes in patients with high and low MYBL1 expression were VHL, PBRM1, TTN, SETD2, and BAP1 (**Figures 4E, F**).

### Immunotherapy response, drug sensitivity and nomogram plot of MYBL1 in ccRCC

**Figure 5:**
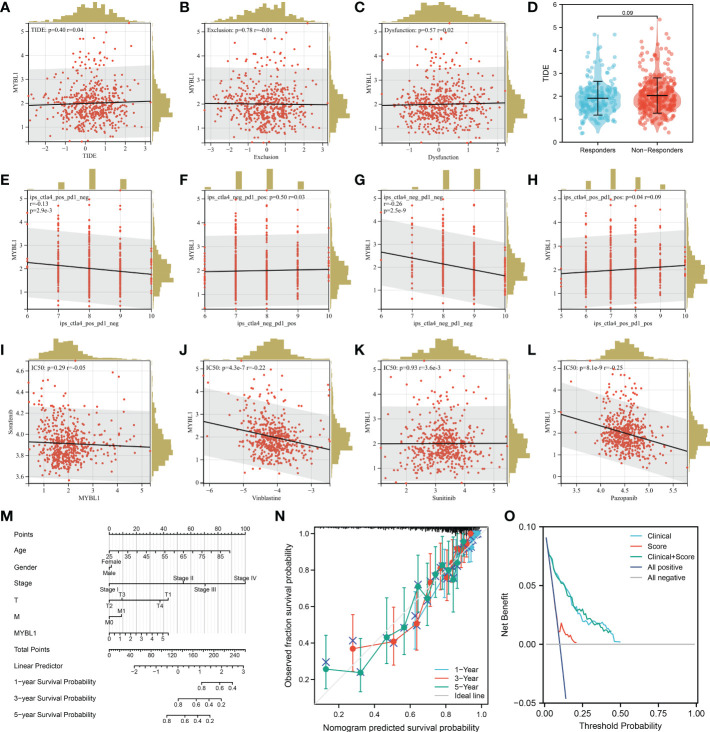
Immunotherapy and drug sensitivity of MYBL1. **(A)** Correlation of MYBL1 with TIDE; **(B)** Correlation of MYBL1 with immune exclusion; **(C)** Correlation of MYBL1 with immune dysfunction; **(D)** The MYBL1 expression in immunotherapy responders and non-responders; **(E–H)** Correlation of MYBL1 with IPS score; **(I–L)** Correlation of MYBL1 and drug sensitivity; **(M)** The nomogram plot based on MYBL and nomogram plot; **(N)** The calibration curve; **(O)** The DCA curve.

We next calculate the TIDE score of each ccRCC patient based on the TIDE analysis. It seems that MYBL1 had no significant influence on the TIDE score, immune exclusion, immune dysfunction quantified by the TIDE analysis (**Figures 5A–D**). Another aspect, a negative correlation was found in MYBL1 with ips_CTLA4_pos_PD1_neg and ips_CTLA4_neg_PD1_pos, indicating that MYBL1 could affect the immunotherapy response of ccRCC patients (**Figures 5E–H**). For the common target drugs for ccRCC, we found that MYBL1 can increase the sensitivity of vinblastine and pazopanib (**Figures 5I–L**). Then, a nomogram plot was constructed by combining the clinical features and MYBL1 expression (**Figure 5M**). The calibration curve indicated that a good fit between actual and nomogram predicted survival (**Figure 5N**). Also, the DCA curves showed that the clinical features can improve the performance of MYBL1 on prognosis prediction (**Figure 5O**).

### MYBL1 enhances the malignant biological behaviors of ccRCC

**Figure 6:**
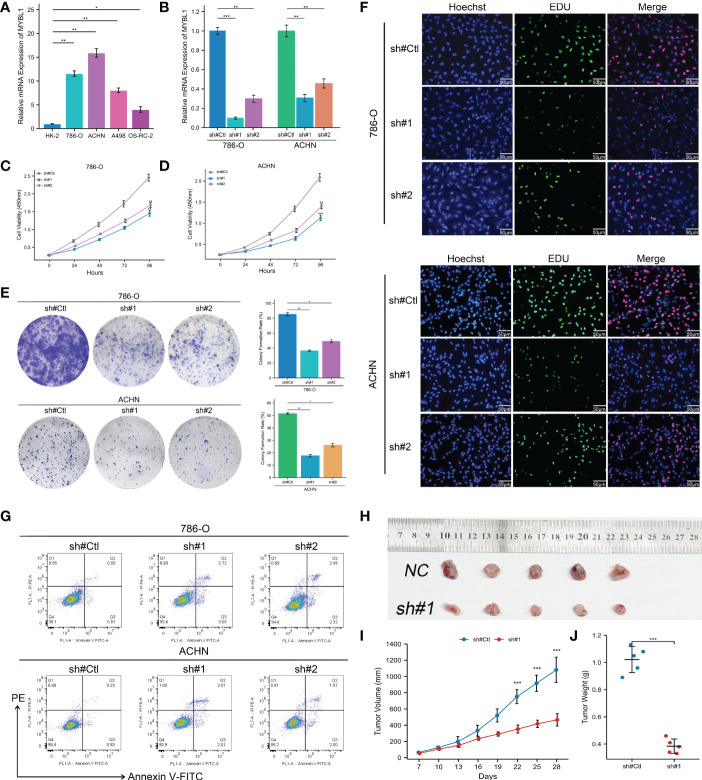
MYBL1 enhances the malignant biological behaviors of ccRCC cells. **(A)** The expression level of MYBL1 in renal cancer cells; **(B)** Knockdown efficiency of MYBL1 in ccRCC cells; **(C, D)** CCK8 assay between sh-MYBL1 and control cells; **(E)** Colony formation assay between sh-MYBL1 and control cells; **(F)** EdU assay between sh-MYBL1 and control cells; **(G)** Flow cytometry detecting cell apoptosis between sh-MYBL1 and control cells; **(H–J)** *In vivo* assay showed that the inhibition of MYBL1 could also hamper tumor growth in mice. *P < 0.05, **P < 0.01, ***P < 0.001.

**Figure 7:**
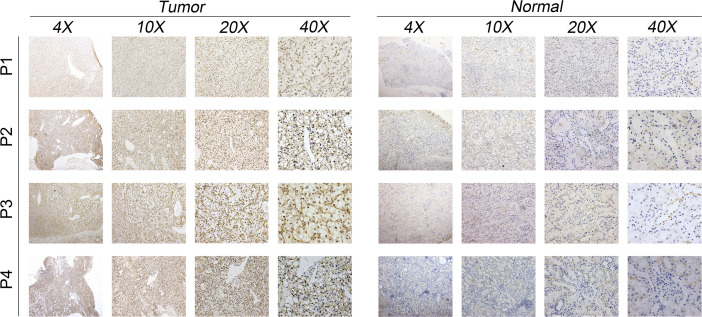
IHC result of MYBL1 between ccRCC tumor and control tissue.

The qRT-PCR result of cell lines indicated that the MYBL1 was overexpressed in ccRCC cells compared to the control cells (**Figure 6A**). Knockdown efficacy of MYBL1 was shown in **Figure 6B**. CCK8 assay indicated that the inhibition of MYBL1 might remarkably increase the proliferation ability of ccRCC cells (**Figures 6C, D**). The same trend was validated by colony formation assay (**Figure 6E**). Meanwhile, we observed a lower percentage of EdU-positive cells in MYBL1 knockdown cells (**Figure 6F**). Flow cytometry results indicated that the knockdown of MYBL1 could remarkably increase the apoptosis rate of ccRCC cells (**Figure 6G**). *In vivo* assay showed that the inhibition of MYBL1 could also hamper tumor growth in mice (**Figures 6H–J**). IHC showed that MYBL1 was overexpressed in ccRCC cancer tissue compared with the normal tissue obtained from four patients (**Figure 7**).

### Machine learning identified the prognosis signature based on MYBL1-derived molecules

**Figure 8:**
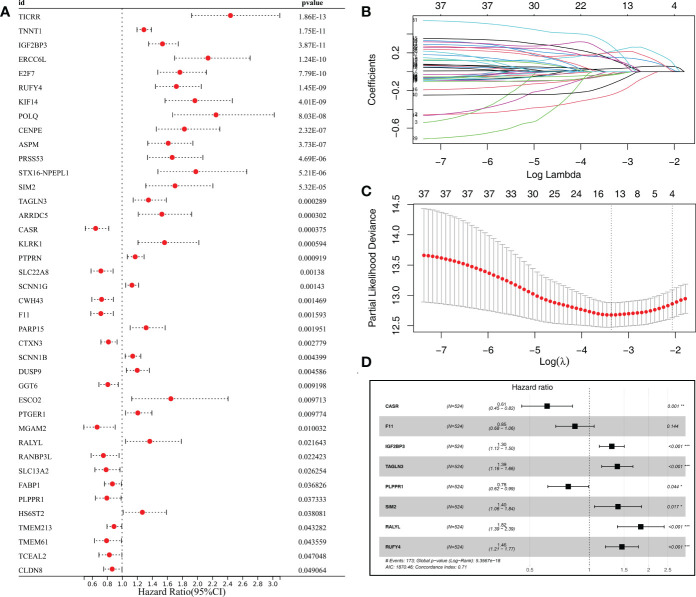
Identification of the prognosis signature based on the machine learning algorithm from MYBL1-derived molecules. **(A)** The top 50 molecules significantly correlated with patients prognosis from MYBL1-derived molecules; **(B, C)** LASSO logistics regression was utilized for data dimension reduction; **(D)** Multivariate cox regression analysis.

**Figure 9:**
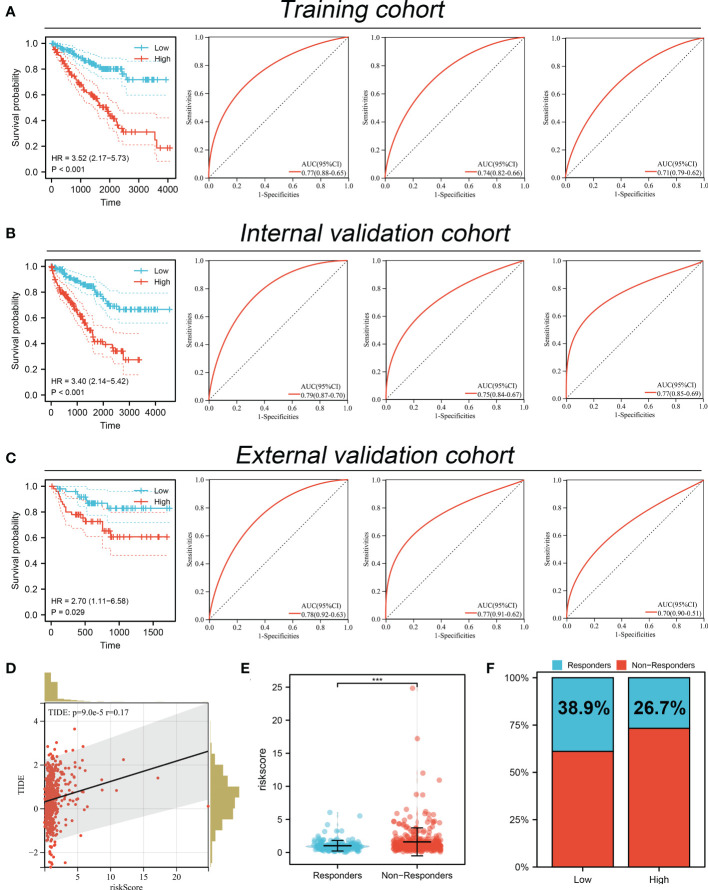
Performance of prognosis signature in predicting patients survival and immunotherapy. **(A)** The performance of our signature in predicting patients survival in the training cohort; **(B)** The performance of our signature in predicting patients survival in the internal validation cohort; **(C)** The performance of our signature in predicting patients survival in external validation cohort; **(D)** Correlation of risk score and TIDE; **(E)** The risk score level in immunotherapy responders and non-responders; **(F)** Percentage of immunotherapy responder in patients with high and low MYBL1 expression. ***P <0.001.

Based on the MYBL1-derived DEGs identified above, univariate Cox regression analysis was conducted to identify the prognosis-related molecules with P < 0.05. The top 50 significant prognosis-related genes were shown in **Figure 8A**. The machine learning algorithm – LASSO logistics regression was utilized to identify the best variable (**Figures 8B, C**). Multivariate Cox regression analysis was utilized to identify a prognosis signature with the formula of “Risk score = CASR * -0.492 + F11 * -0.167 + IGF2BP3 * 0.262 + TAGLN3 * 0.327 + PLPPR1 * -0.245 + SIM2 * 0.334 + RALYL * 0.601 + RUFY4 * 0.381” (**Figure 8D**). In the training cohort, the patients in the high risk group might have a worse OS (**Figure 9A**). Also, our signature showed a good prediction ability in patients survival (**Figure 9A**; 1-year AUC = 0.77; 3-year AUC = 0.74, 5-year AUC = 0.71). Meanwhile, the satisfactory performance of our signature was also observed in the internal validation and external validation cohort (**Figures 9B, C**). Next, we noticed a positive correlation between the risk score and TIDE score (**Figure 9D**, R = 0.17, P < 0.001). We found that the immunotherapy responders defined by TIDE analysis tend to have a higher risk score level (**Figure 9E**). Also, the percentage of immunotherapy responders in high risk patients was 26.7%, greatly lower than 38.9% in low risk patients (**Figure 9F**). GSEA analysis indicated that the in high risk patients, the pathway of pancreas beta cells, allograft rejection, KRAS signaling, IL6/JAK/STAT3 signaling, spermatogenesis, E2F targets, G2M checkpoints, angiogenesis were significantly activated (**Figure S2**).

## Discussion

Renal cancer is still a threatening disease globally and responsible for almost 2 million deaths per year, resulting in a great public burden (25). Although surgical treatment can provide reliable prognosis benefits for early patients, the survival performance of advanced patients is still limited. Consequently, it is extremely meaningful to identify novel biomarkers for ccRCC diagnosis and therapy options.

Our study examined the role of MYBL1 in ccRCC in depth. According to our knowledge, this is the first study investigating MYBL1 in ccRCC. Here, through a series of bioinformatics analysis, we found that MYBL1 was highly expressed in ccRCC patients in both RNA and protein levels. Prognosis analysis revealed that MYBL1 was correlated with poor prognosis performance, including OS, DSS and PFI. Clinical correlation analysis showed that MYBL1 was higher in the worse N- and M-stage. Furthermore, biological enrichment analysis was conducted to explore the biological role of MYBL1 in ccRCC. Moreover, we found that MYBL1 can remodel the immune microenvironment of ccRCC and affect the immunotherapy response. *In vitro* and *in vivo* assays indicated that MYBL1 was upregulated in ccRCC cells and can promote cellular malignant behaviors of ccRCC. Meanwhile, the prognosis signature based on the MYBL1-derived molecules showed great prediction ability on patient prognosis in both training and validation cohorts.

We found that MYBL1 is a risk factor for ccRCC based on bioinformatic analysis and experiments. Also, biological enrichment analysis indicated that MYBL1 was mainly enriched in the terms of the inflammatory response, G2M checkpoint and E2F targets. Batova et al. indicated that the acute inflammatory response could be regulated by Englerin A, therefore changing the cell metabolism level and affecting renal cancer progression (26). During the cell cycle, the G2/M checkpoint is an important step. Ding et al. indicated that the dioscin could hamper cell proliferation of osteosarcoma cells based on a G2/M checkpoint-dependent manner (27). Meanwhile, Kent et al. revealed that the dysfunction of E2F in cancers tends to induce the carcinogenic cascade reaction (28). Our result indicated that the MYBL1 might enhance ccRCC progression by affecting the activity of the above pathways.

The immune microenvironment can influence cancer development through complex biological interactions. In our study, we found that the MYBL1 MYBL1 can increase Tregs, M2 macrophages, neutrophils, B cells, monocytes, CD8+ T cells, yet decrease endothelial cells level in the ccRCC microenvironment. Tregs can result in an inhibitory immune microenvironment. In ccRCC, Ji et al. found that the hamper of Tregs in the colon cancer microenvironment can improve the anti-tumor effect and inhibit cancer metastasis (29). Li et al. indicated that aiduqing formula can inhibit Treg infiltration induced by TAM/CXCL1, further hampering breast cancer metastasis (30). In solid tumors, M2 macrophages are generally cancer promoters (31). Chen et al. revealed that gastric and breast cancer metastasis can be facilitated by M2 macrophages recruited by the local tumor microenvironment based on secreted CHI3L1 (32). Xie et al. revealed that the CXCL13 secreted by M2 macrophages facilitated the metastatic potential of ccRCC (33). Based on a comprehensive review conducted by Xiong et al., carcinogenesis and metastasis of cancer can be facilitated by neutrophils (34). These results indicate that MYBL1 might be immune-related molecules that can remodel the immune microenvironment of ccRCC patients.

Moreover, we established a prognosis signature based on the MYBL1-derived molecules. Our signature presents a good prediction ability on patient survival performance. Moreover, the ccRCC patients in different groups might have different responses to immunotherapy. These results indicated the clinical application value of MYBL1 in the clinical.

Although our research is based on high-quality analysis and rigorous experiments, some limitations still need to be noted. Firstly, the population used for analysis was mainly Western. The biological difference between different races can decrease the reliability of our conclusions. Secondly, the in-deep mechanism of MYBL1 to enhance the cellular malignant behaviors of ccRCC is still unclear. In the future, more basic studies focused on MYBL1 in cancers, especially in ccRCC, are needed.

## Data availability statement

The original contributions presented in the study are included in the article/**Supplementary Material**. Further inquiries can be directed to the corresponding author.

## Ethics statement

The studies involving human participants were reviewed and approved by Medical Ethics Committee of First Affiliated Hospital of Harbin Medical University. The patients/participants provided their written informed consent to participate in this study. The animal study was reviewed and approved by Animal Care and Use Committee of the First Affiliated Hospital of Harbin Medical University.

## Author contributions

TW and WJ performed the public data analysis. TW and WX wrote the manuscript. TW and WJ performed the experiments. CZ designed this work. All authors contributed to the article and approved the submitted version.

## Supplementary material

Supplementary Figure 1 The flow chart of our study

Supplementary Figure 2 GSEA analysis based on Hallmark gene set between high and low risk patients.

## References

[1] Capitanio U Montorsi F. Renal cancer. Lancet (London England) (2016) 387:894–906. doi: 10.1016/s0140-6736(15)00046-x 26318520

[2] Wolf MM Kimryn Rathmell W Beckermann KE. Modeling clear cell renal cell carcinoma and therapeutic implications. Oncogene (2020) 39:3413–26. doi: 10.1038/s41388-020-1234-3 PMC7194123 32123314

[3] Sato Y Yoshizato T Shiraishi Y Maekawa S Okuno Y Kamura T. Integrated molecular analysis of clear-cell renal cell carcinoma. Nat Genet (2013) 45:860–7. doi: 10.1038/ng.2699 23797736

[4] Jonasch E Gao J Rathmell WK. Renal cell carcinoma. BMJ (Clinical Res ed.) (2014) 349:g4797. doi: 10.1136/bmj.g4797 PMC4707715 25385470

[5] Yin Z Wu D Shi J Wei X Jin N Lu X. Identification of ALDH3A2 as a novel prognostic biomarker in gastric adenocarcinoma using integrated bioinformatics analysis. BMC Cancer (2020) 20:1062. doi: 10.1186/s12885-020-07493-x 33148208 PMC7640415

[6] Wei Y Chen X Ren X Wang B Zhang Q Bu H. Identification of MX2 as a novel prognostic biomarker for sunitinib resistance in clear cell renal cell carcinoma. Front Genet (2021) 12:680369. doi: 10.3389/fgene.2021.680369 34306023 PMC8299280

[7] Zhu J Wu Y Yu Y Li Y Shen J Zhang R. MYBL1 induces transcriptional activation of ANGPT2 to promote tumor angiogenesis and confer sorafenib resistance in human hepatocellular carcinoma. Cell Death Dis (2022) 13:727. doi: 10.1038/s41419-022-05180-2 35987690 PMC9392790

[8] Guo H Zhang B Nairn AV Nagy T Moremen KW Buckhaults P. O-Linked n-acetylglucosamine (O-GlcNAc) expression levels epigenetically regulate colon cancer tumorigenesis by affecting the cancer stem cell compartment *via* modulating expression of transcriptional factor MYBL1. J Biol Chem (2017) 292:4123–37. doi: 10.1074/jbc.M116.763201 PMC5354504 28096468

[9] Brayer KJ Frerich CA Kang H Ness SA. Recurrent fusions in MYB and MYBL1 define a common, transcription factor-driven oncogenic pathway in salivary gland adenoid cystic carcinoma. Cancer Discov (2016) 6:176–87. doi: 10.1158/2159-8290.Cd-15-0859 PMC4744535 26631070

[10] Ramkissoon LA Horowitz PM Craig JM Ramkissoon SH Rich BE Schumacher SE. Genomic analysis of diffuse pediatric low-grade gliomas identifies recurrent oncogenic truncating rearrangements in the transcription factor MYBL1. Proc Natl Acad Sci United States America (2013) 110:8188–93. doi: 10.1073/pnas.1300252110 PMC3657784 23633565

[11] Nikolaus M Koch A Stenzel W Elezkurtaj S Sahm F Tietze A. Atypical NMDA receptor expression in a diffuse astrocytoma, MYB- or MYBL1-altered as a trigger for autoimmune encephalitis. Acta Neuropathol (2022) 144:385–9. doi: 10.1007/s00401-022-02447-y PMC9288378 35727368

[12] Bindea G Mlecnik B Hackl H Charoentong P Tosolini M Kirilovsky A. ClueGO: a cytoscape plug-in to decipher functionally grouped gene ontology and pathway annotation networks. Bioinf (Oxford England) (2009) 25:1091–3. doi: 10.1093/bioinformatics/btp101 PMC2666812 19237447

[13] Yu G Wang LG Han Y He QY. clusterProfiler: an r package for comparing biological themes among gene clusters. Omics J Integr Biol (2012) 16:284–7. doi: 10.1089/omi.2011.0118 PMC3339379 22455463

[14] Subramanian A Tamayo P Mootha VK Mukherjee S Ebert BL Gillette MA. Gene set enrichment analysis: a knowledge-based approach for interpreting genome-wide expression profiles. Proc Natl Acad Sci United States America (2005) 102:15545–50. doi: 10.1073/pnas.0506580102 PMC1239896 16199517

[15] Ren X Chen X Zhang X Jiang S Zhang T Li G. Immune microenvironment and response in prostate cancer using Large population cohorts. Front Immunol (2021) 12:686809. doi: 10.3389/fimmu.2021.686809 34777331 PMC8585452

[16] Chen B Khodadoust MS Liu CL Newman AM. & alizadeh, a. a. profiling tumor infiltrating immune cells with CIBERSORT. Methods Mol Biol (Clifton NJ) (2018) 1711:243–59. doi: 10.1007/978-1-4939-7493-1_12 PMC5895181 29344893

[17] Li T Fan J Wang B Traugh N Chen Q Liu JS. TIMER: A web server for comprehensive analysis of tumor-infiltrating immune cells. Cancer Res (2017) 77:e108–10. doi: 10.1158/0008-5472.Can-17-0307 PMC6042652 29092952

[18] Becht E Giraldo NA Lacroix L Buttard B Elarouci N Petitprez F. Estimating the population abundance of tissue-infiltrating immune and stromal cell populations using gene expression. Genome Biol (2016) 17:218. doi: 10.1186/s13059-016-1070-5 27765066 PMC5073889

[19] Plattner C Finotello F. & rieder, d. deconvoluting tumor-infiltrating immune cells from RNA-seq data using quanTIseq. Methods Enzymol (2020) 636:261–85. doi: 10.1016/bs.mie.2019.05.056 32178821

[20] Van Allen EM Miao D Schilling B Shukla SA Blank C Zimmer L. Genomic correlates of response to CTLA-4 blockade in metastatic melanoma. Sci (New York NY) (2015) 350:207–11. doi: 10.1126/science.aad0095 PMC5054517 26359337

[21] Jiang P Gu S Pan D Fu J Sahu A Hu X. Signatures of T cell dysfunction and exclusion predict cancer immunotherapy response. Nat Med (2018) 24:1550–8. doi: 10.1038/s41591-018-0136-1 PMC6487502 30127393

[22] Malta TM Sokolov A Gentles AJ Burzykowski T Poisson L Weinstein JN. Machine learning identifies stemness features associated with oncogenic dedifferentiation. Cell (2018) 173:338–354.e315. doi: 10.1016/j.cell.2018.03.034 29625051 PMC5902191

[23] McNeish DM. Using lasso for predictor selection and to assuage overfitting: A method long overlooked in behavioral sciences. Multivariate Behav Res (2015) 50:471–84. doi: 10.1080/00273171.2015.1036965 26610247

[24] Yu Y Yao W Wang T Xue W Meng Y Cai L. FBXL6 depletion restrains clear cell renal cell carcinoma progression. Trans Oncol (2022) 26:101550. doi: 10.1016/j.tranon.2022.101550 PMC9526225 36183674

[25] Morris MR Latif F. The epigenetic landscape of renal cancer. Nat Rev Nephrol (2017) 13:47–60. doi: 10.1038/nrneph.2016.168 27890923

[26] Batova A Altomare D Creek KE Naviaux RK Wang L Li K. Englerin a induces an acute inflammatory response and reveals lipid metabolism and ER stress as targetable vulnerabilities in renal cell carcinoma. PloS One (2017) 12:e0172632. doi: 10.1371/journal.pone.0172632 28296891 PMC5351975

[27] Ding Q Zhang W Cheng C Mo F Chen L Peng G. Dioscin inhibits the growth of human osteosarcoma by inducing G2/M-phase arrest, apoptosis, and GSDME-dependent cell death *in vitro* and *in vivo* . J Cell Physiol (2020) 235:2911–24. doi: 10.1002/jcp.29197 31535374

[28] Kent LN Leone G. The broken cycle: E2F dysfunction in cancer. Nat Rev Cancer (2019) 19:326–38. doi: 10.1038/s41568-019-0143-7 31053804

[29] Ji D Song C Li Y Xia J Wu Y Jia J. Combination of radiotherapy and suppression of tregs enhances abscopal antitumor effect and inhibits metastasis in rectal cancer. J Immunother Cancer (2020) 8(2):e000826. doi: 10.1136/jitc-2020-000826 33106387 PMC7592256

[30] Li J Wang S Wang N Zheng Y Yang B Wang X. Aiduqing formula inhibits breast cancer metastasis by suppressing TAM/CXCL1-induced treg differentiation and infiltration. Cell Communication Signaling CCS (2021) 19:89. doi: 10.1186/s12964-021-00775-2 34461944 PMC8404313

[31] Mantovani A Sozzani S Locati M Allavena P Sica A. Macrophage polarization: tumor-associated macrophages as a paradigm for polarized M2 mononuclear phagocytes. Trends Immunol (2002) 23:549–55. doi: 10.1016/s1471-4906(02)02302-5 12401408

[32] Chen Y Zhang S Wang Q Zhang X. Tumor-recruited M2 macrophages promote gastric and breast cancer metastasis *via* M2 macrophage-secreted CHI3L1 protein. J Hematol Oncol (2017) 10:36. doi: 10.1186/s13045-017-0408-0 28143526 PMC5286803

[33] Xie Y Chen Z Zhong Q Zheng Z Chen Y Shangguan W. M2 macrophages secrete CXCL13 to promote renal cell carcinoma migration, invasion, and EMT. Cancer Cell Int (2021) 21:677. doi: 10.1186/s12935-021-02381-1 34922542 PMC8684162

[34] Xiong S Dong L Cheng L. Neutrophils in cancer carcinogenesis and metastasis. J Hematol Oncol (2021) 14:173. doi: 10.1186/s13045-021-01187-y 34674757 PMC8529570

