# Supplementary figures and images for: Integration analysis identifies MYBL1 as a novel immunotherapy biomarker affecting the immune microenvironment in clear cell renal cell carcinoma: Evidence based on machine learning and experiments
Source: Front Immunol. 2022 Dec 14;13:1080403. doi: 10.3389/fimmu.2022.1080403 (PMC9794576; doi:10.3389/fimmu.2022.1080403)

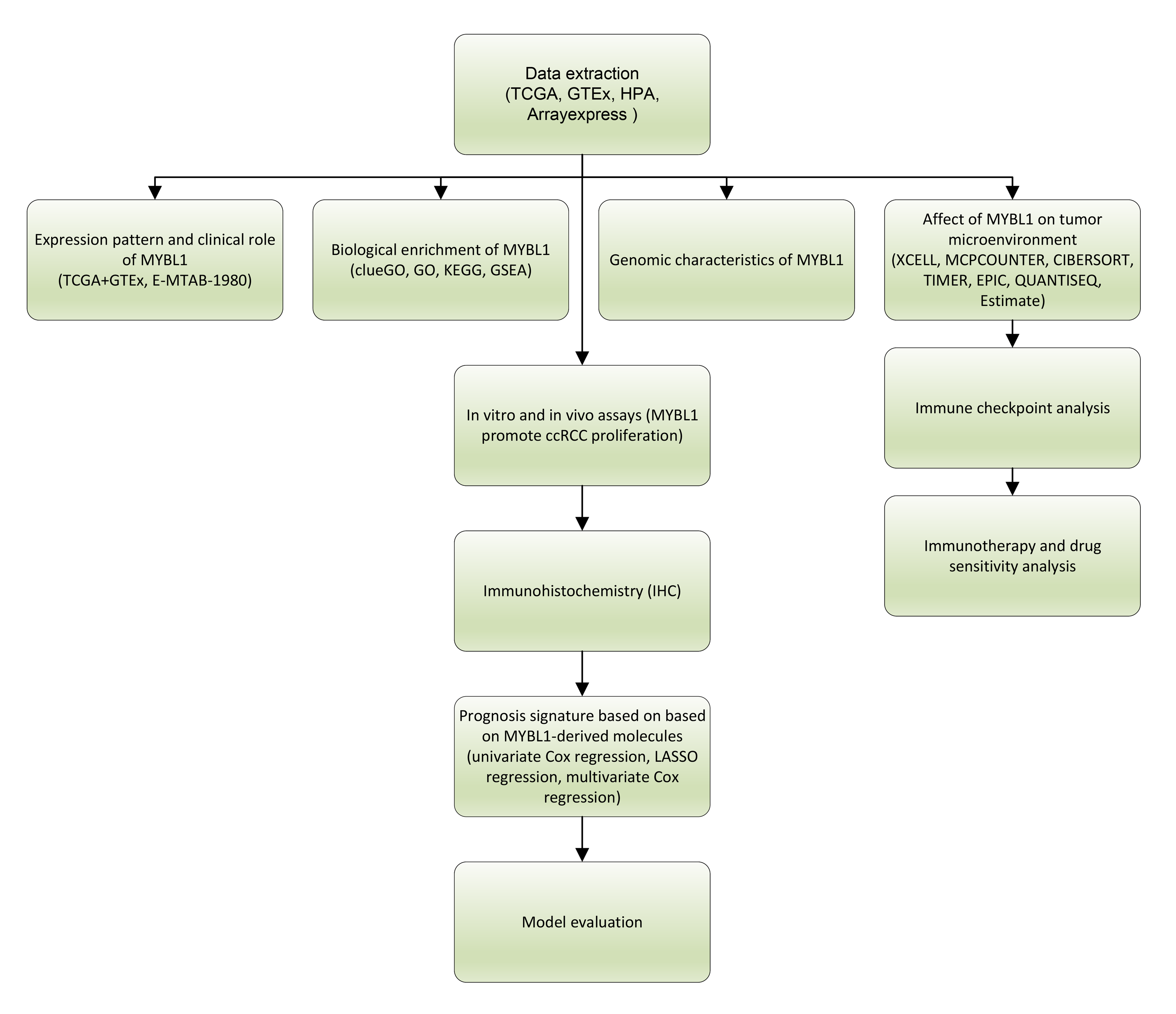

Supplement: Supplementary Figure 1 — The flow chart of our study [file Image_1.tif]

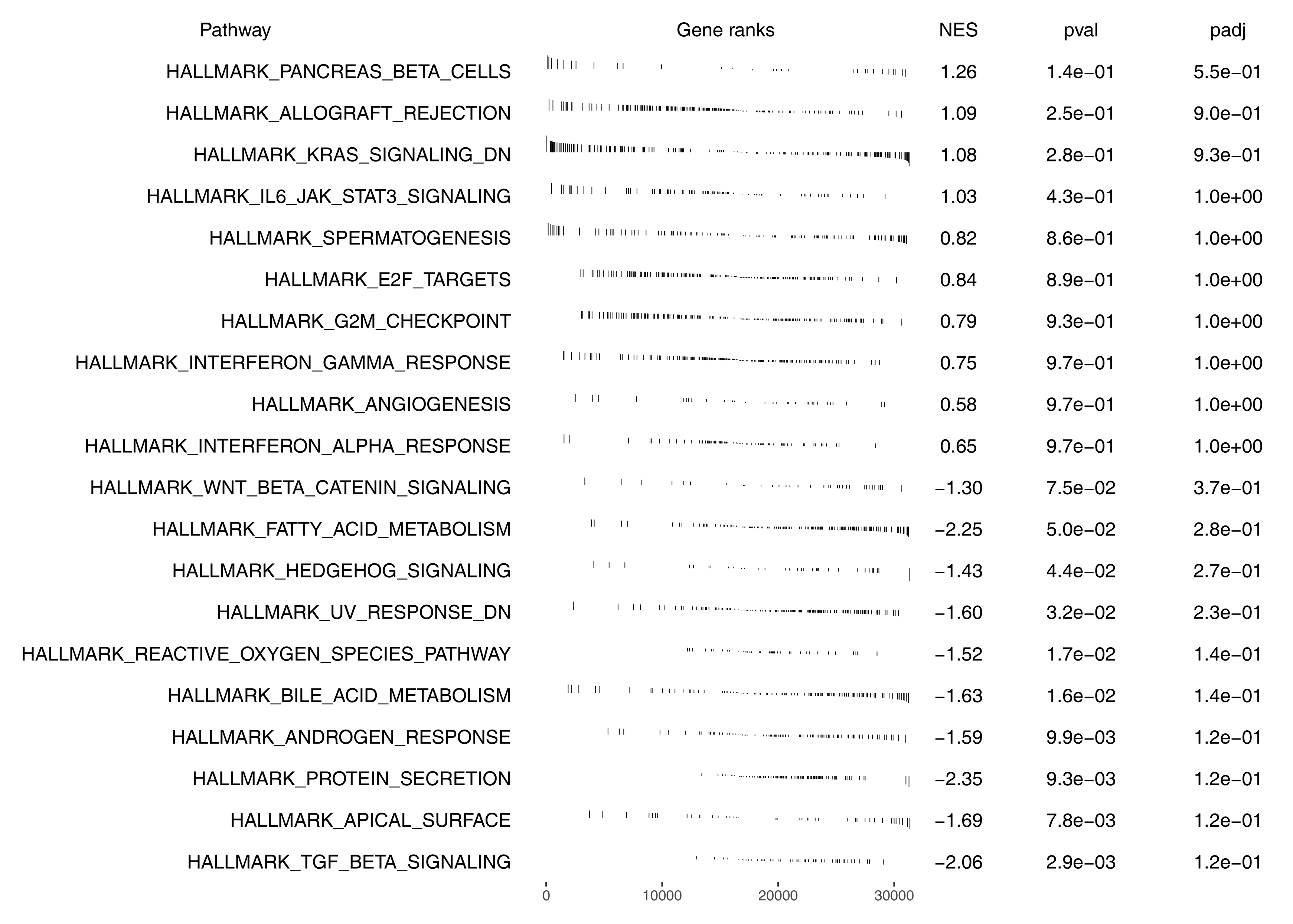

Supplement: Supplementary Figure 2 — GSEA analysis based on Hallmark gene set between high and low risk patients. [file Image_2.tif]
